# UHPLC-UV Method for Simultaneous Determination of Perindopril Arginine and Indapamide Hemihydrate in Combined Dosage Form: A Stability-Indicating Assay Method

**DOI:** 10.3390/scipharm86010007

**Published:** 2018-02-22

**Authors:** Naser F. Al-Tannak

**Affiliations:** Department of Pharmaceutical Chemistry, Faculty of Pharmacy, Kuwait University, P.O. Box 23924, Safat, 13110 Kuwait City, Kuwait; dr_altannak@hsc.edu.kw; Tel.: +965-2463-6070

**Keywords:** Coversyl Plus^®^, Perindopril arginine, Indapamide hemihydrate, UHPLC, stability indicating, forced degradation studies, solid phase extraction, UHPLC-QToF-MS

## Abstract

Perindopril arginine and Indapamide hemihydrate in combination were proven to have a synergistic antihypertensive impact when compared with the use of each component alone. Therefore, a new Ultra-High Performance Liquid Chromatography coupled with Ultraviolet detector (UHPLC-UV) method has been developed and subsequently validated for simultaneous determination of the anti-hypertensive combination of Perindopril arginine and Indapamide hemihydrate. The separation of Perindopril arginine and Indapamide hemihydrate was achieved using a BEH C18 (1.7 μm, 2.1 × 50 mm) analytical column (Waters^®^ Acquity UPLC) and a mobile phase composed of 0.01% v/v formic acid in water adjusted to pH 4 with acetic acid and acetonitrile (40:60 v/v). The method was able to separate Perindopril arginine and Indapamide hemihydrate within less than 4.5 min with high accuracy, precision, resolution, and sensitivity. The content of Perindopril arginine and Indapamide hemihydrate present in the dosage form Coversyl Plus^®^ (5000 µg of Perindopril arginine/1250 µg of Indapamide hemihydrate) was determined in triplicate to give a concentration of 4991 µg and 1247 µg, respectively, from the manufacturer’s stated amounts with Relative Standard Deviation (%RSD) of ±0.63% for Perindopril arginine and ±0.84% for Indapamide hemihydrate. Moreover, the degradation products of the combination were elucidated by UHPLC-Quadrupole Time of Flight-Mass spectrometry (UHPLC-QToF-MS) under acidic, basic, and thermal conditions. In conclusion, the developed UHPLC-UV method was sensitive, rapid, and precise. Furthermore, forced degradation studies were performed and the degradants were identified by UHPLC-Electro-Spray Ionization-QToF (UHPLC-ESI-QToF).

## 1. Introduction

Hypertension is one of the worldwide leading causes of mortality. It is a major adjustable risk factor for cardiovascular disease and stroke [1]. Different classes of medicines can be used to reduce blood pressure [2]. However, monotherapy as a treatment for hypertension can successfully reduce blood pressure in few patients. Therefore, the majority will necessitate treatment with two or more drugs to reach normal levels. The main aim of using combination therapy is to utilize the various mechanisms of action, which have the potential to control blood pressure due to the combined effects, as well as to lower the doses of both drugs to diminish unwanted side effects [1].

**Figure 1:**
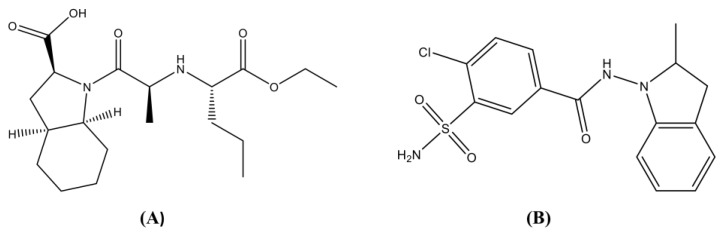
Chemical structures for (**A**) Perindopril and (**B**) Indapamide.

As shown in Figure 1A, Perindopril is a long-acting angiotensin-converting enzyme (ACE) inhibitor. Taken once daily, Perindopril (4000 to 8000 µg) is effective at controlling blood pressure in patients with mild to moderate hypertension. Patients who do not respond effectively to Perindopril as a monotherapy usually respond with the addition of a second antihypertensive agent [3]. As clearly shown in Figure 1B, Indapamide, is a sulfonamide derivative pharmacologically related to thiazide diuretics [4]. In spite of reports that proved that the antihypertensive activity of Indapamide is mainly due to its diuretic activity, few cases of diuresis were discovered with the typical antihypertensive dose of 2500 µg daily [4]. Various studies suggest that it may also lower blood pressure by lowering vascular reactivity and peripheral vascular resistance [4]. Indapamide has been successfully reported to control mild to moderate hypertension as a monotherapy or in combination with other antihypertensive agents [5].

The gainful impacts of Indapamide and Perindopril as a combination therapy in reducing blood pressure have been demonstrated in numerous trials [4,5,6].

Perindopril arginine and Indapamide hemihydrate as a combination therapy was proven to have a major effect on systolic blood pressure, arterial stiffness, and microcirculatory alterations [4,5,6,7]. Thus, different studies and pharmaceutical industries are competing to produce different combinations of Perindopril arginine and Indapamide hemihydrate to keep patients’ blood pressure within an acceptable range. Coversyl Plus^®^ (Servier, Wexham, UK) is a new pharmaceutical product that contains 5000 µg of Perindopril arginine and 1250 µg of Indapamide hemihydrate to control blood pressure in hypertensive patients [8]. Therefore, a rapid and efficient analytical method is required to qualitatively and quantitatively indicate the amounts of Perindopril arginine and Indapamide hemihydrate in its combined pharmaceutical form.

A literature survey revealed that Perindopril is officially in the *British Pharmacopoeia* [9], whereas Indapamide is officially in the *British Pharmacopoeia* [10] and *U.S. Pharmacopeia* (USP) [11]. A detailed literature survey found that a number of reported methods have been constructed for the estimation of Perindopril arginine and Indapamide hemihydrate individually [11,12,13,14,15,16,17,18,19,20,21,22].

There are few methods reported in the literature for the simultaneous determination of Perindopril and Indapamide in combined dosage form, including High-Performance Thin-Layer Chromatography (HPTLC) [23], and Ultraviolet instrument (UV) spectrophotometric determination of the two drugs by the simultaneous equation method [24]. High-Performance Liquid Chromatography (HPLC) and two spectrophotometric methods have been also developed for resolving a binary mixture of the two drugs in their pharmaceutical dosage forms [25]. Moreover, one stability-indicating reverse phase (RP)-HPLC method has been reported for the combination [26]. However, the reported methods had many disadvantages such as complex mobile phase system [23], UV where the chromatographic separation was not applicable [23,24], as well as using a phosphate buffer as an aqueous phase with a low pH, which is incompatible with a mass spectrometer and has a low pH that can affect the column condition [25,26].

After research, the simultaneous determination of Perindopril arginine and Indapamide hemihydrate in combined pharmaceutical dosage form by Ultra high Performance Liquid Chromatography (UHPLC)-UV has not been reported. Therefore, the current study was proposed to investigate the amount of Perindopril and Indapamide in a combined pharmaceutical dosage form called Coversyl Plus^®^, using an UHPLC-UV instrument as well as forced degradation studies on the combination to identify degradant products and degradation pathways by UHPLC-Electro-Spray Ionization-Quadrupole Time-of-Flight mass spectrometer (UHPLC-ESI-QToF). Therefore, we claim that the developed method can be considered a stability-indicating method.

## 2. Materials and Methods

### 2.1. Materials

Perindopril arginine (98.0%) was provided by ALSACHIM (Illkirch Graffenstaden, France). Indapamide hemihydrate (≥97.0%) reference standard was kindly provided by the Egyptian Drug Authority (Cairo, Egypt). Formic acid (≥96.0%) and HPLC-grade acetonitrile were purchased from Sigma-Aldrich (St. Louis, MO, USA). A Coversyl Plus^®^ tablet containing 5000 µg of Perindopril arginine and 1250 µg Indapamide hemihydrate was obtained from a local market in Egypt. Acetic acid (99–100%) was supplied by Avantor Performance Materials (Center Valley, PA, USA). HPLC-grade water was prepared “in house” with a MilliQfilter (Millipore, Watford, UK). Nylon solvent filters (0.45 µm) (Waters^®^, Elstree, UK).

### 2.2. Instruments

#### 2.2.1. UHPLC Instrumentation

Waters^®^ Acquity UPLC system with quaternary Solvent Manager (H-Class), Sample Manager and UV detector, Waters^®^ Acquity UPLC BEH C18, 1.7 µm, 2.1 × 50 mm analytical column were used for the analysis and method validation. Empower^®^ software was used for data processing and reporting.

#### 2.2.2. Liquid Chromatography–Mass Spectrometry (LC-MS)

Waters^®^ Xevo G2-S QToF coupled with Waters^®^ Acquity UPLC system with binary Solvent Manager (I-Class) via ESI interface. The operating parameters in the positive ion mode were as follows: the sheath gas and auxiliary flow rates were set at 30 and 5 (arbitrary unit), respectively. The capillary voltage was set at 3.5 V, sampling cone was 55 V and source temperature was 110 °C. Source temperature and desolvation temperature were set at 110 and 450 °C, respectively. Collision energy 2: 10 eV 3: 15 eV 4: 20 eV.

### 2.3. Chromatographic Conditions

Isocratic elution was carried out with a mobile phase comprised of filtered and degassed 0.01% v/v formic acid in water adjusted to pH 4 with acetic acid and acetonitrile in a 40:60 v/v proportion and pumped at a flow rate of 0.3 mL/min. The column temperature was set at 25 °C and samples were analyzed at a wavelength of 227 nm and injected at 1 µL injection volume. 

### 2.4. Standard Solutions of Perindopril Arginine and Indapamide Hemihydrate 

Primary standard stock solutions of Perindopril arginine and Indapamide hemihydrate (all 10.000 µg/mL) were separately prepared by dissolving 100.000 µg of each standard powder in a 10-mL volumetric flask using acetonitrile. Primary stock solutions were diluted with the mobile phase to prepare standard working solutions of each (1000 µg/mL). All solutions were stored at 4 °C and equilibrated to room temperature before use.

### 2.5. Validation

Validation of the method was performed according to The International Council for Harmonisation (ICH) guidelines [27].

#### 2.5.1. System Suitability Test

A system suitability test was established from three replicate injections of a solution containing 30 µg/mL of Perindopril arginine and Indapamide hemihydrate. The peak tailing for the drug was measured. A useful and practical measurement of peak shape, peak tailing, and theoretical plate count was determined. Column plate number was determined using the formula N = 5.54 (T_R_/W_h_)^2^, where T_R_ is the peak retention time and W_h_ is the bandwidth at 50% of peak height.

#### 2.5.2. Linearity and Calibration Curves

Accurately measured aliquots of Perindopril arginine and Indapamide hemihydrate were transferred from their working standard solution (1000 µg/mL) into a series of 10-mL volumetric flasks and filled to volume with the mobile phase. The calibration samples consisted of five concentrations of Perindopril arginine (20–450 µg/mL) and five concentrations of Indapamide hemihydrate (15–112.5 µg/mL). The samples were injected separately into the BEH C18 column under a flow rate of 0.3 mL/min. The peak area of each drug was recorded against its concentration, the linearity of the curves was constructed, and regression equations were computed.

#### 2.5.3. Accuracy

The accuracy of the results was determined by calculating the accuracy (%) of three replicates of three different concentrations covering the linearity. The concentrations were calculated from the corresponding regression equations.

#### 2.5.4. Precision

The precision of the UHPLC method for the combination was evaluated by preparing six sets of the mixture in the concentration ranges of the calibration curve. Precision was evaluated by preparing three different concentrations of pure standards of the cited drugs (20, 250, and 450 μg/mL; 15, 62.5, and 112.5 μg/mL) for Perindopril arginine and Indapamide hemihydrate within the linear range; samples were analyzed in triplicate, on a single day and three consecutive days, to calculate the intra-day (repeatability) and inter-day precision (intermediate precision) of the proposed method, respectively. A set (*n* = 3) was prepared at room temperature (22 °C), while five other sets (*n* = 3) were prepared and stored at 4 °C for mixtures dissolved in mobile phase samples for three days. Percentage relative standard deviation (%RSD) was used to calculate the intra- and inter-assay precision.

#### 2.5.5. Limit of Detection (LOD) and Limit of Quantification (LOQ)

Stock solutions of Perindopril arginine and Indapamide hemihydrate were prepared at concentrations of 1–100 µg/mL. The LOD and LOQ for Perindopril arginine and Indapamide hemihydrate were determined at a signal-to-noise ratio of 3:1 and 10:1, respectively. 

### 2.6. Application to Pharmaceutical Formulation

Five tablets of Coversyl Plus^®^ were powdered and transferred into a 100-mL conical flask; 100 mL of acetonitrile were added and the solution was sonicated for 30 min, then placed on a shaker set to 70 rotations per min for 10 min. The volume was completed to the mark and then filtered, and 1-mL of filtered solution was placed in 2-mL glass screw thread vials for analysis. The concentrations of Perindopril arginine and Indapamide hemihydrate were calculated from the computed regression equations.

### 2.7. Forced Degradation Studies

All stability tests were done on the pharmaceutical formulation to simulate the actual conditions to which the dosage form will be exposed during storage.

#### 2.7.1. Acidic Degradation

One thousand micrograms of the powdered dosage form were weighed and placed in a 4-mL vial. Two milliliters of 1 N HCl were added and heated at 90 °C for 90 min, allowed to cool down for 15 min, and then analyzed by LC-MS.

#### 2.7.2. Basic Degradation

One thousand micrograms of the powdered dosage form were weighed and placed in a 4-mL vial. Two milliliters of 1 N NaOH was added and heated at 90 °C for 90 min, allowed to cool down for 15 min, and then analyzed by LC-MS.

#### 2.7.3. Thermal Degradation

One thousand micrograms of the powdered dosage form were weighed, placed in a 4-mL vial, and heated in an oven at 90 °C for 90 min, allowed to cool down for 15 min, and then analyzed by LC-MS.

## 3. Results and Discussion

The present work considers the first UHPLC-UV method for simultaneous determination of Perindopril arginine and Indapamide hemihydrate. Moreover, the stability of the combination was evaluated under hydrolytic and thermal conditions and the structures of the degradation products were elucidated by LC-ESI-QToF.

### 3.1. Method Development

**Figure 2:**
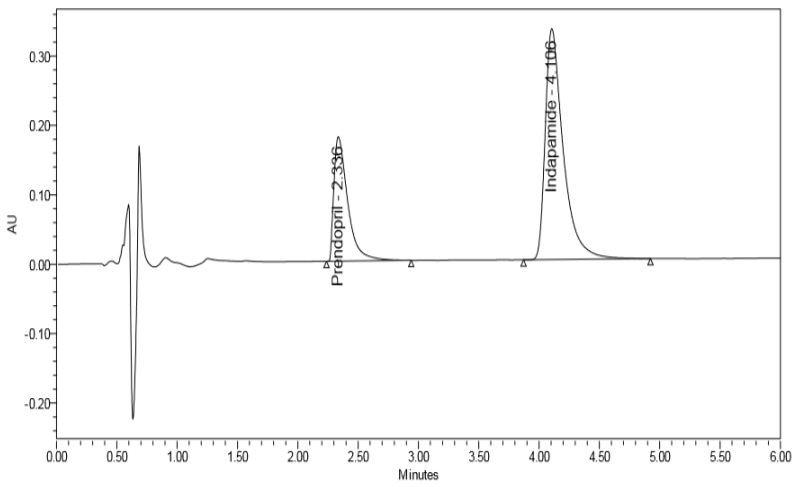
Complete resolution of Perindopril arginine and Indapamide Hemihydrate.

Different organic modifiers proportions, and various buffers with different pH values (3, 5, and 7) were tried. As shown in Figure 2, the optimum resolution and peak shape were obtained with 0.01% v/v formic acid in water, adjusted to pH 4 with acetic acid/acetonitrile (40:60 v/v) as a mobile phase. The flow rate for better resolution and rapid separation was adjusted to 0.3 mL/min. Moreover, the system suitability parameters were calculated for the adopted chromatographic method and presented in Table 1.

A system suitability test was established from three replicate injections of a solution containing 30 µg/mL of Perindopril arginine and Indapamide hemihydrate. The %RSD of the peak area was calculated. The peak tailing of the drug was measured. A useful and practical measurement of peak shape, peak tailing, and theoretical plate count was determined. Column plate number was determined using the formula N = 5.54 (T_R_/W_h_)^2^, where W_h_ is the bandwidth at 50% of peak height. The proposed method met these requirements within the USP-accepted limits (tailing factor ≤ 2; theoretical plates > 2000) [27]. 

### 3.2. Calibration Curves

**Figure 3:**
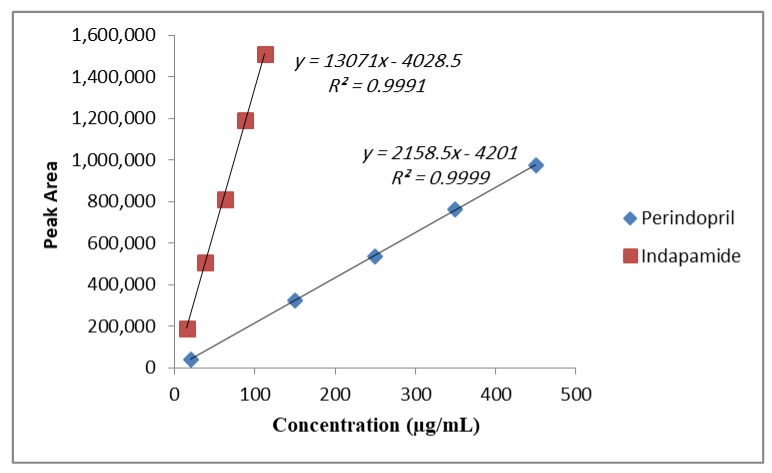
Calibration curve for Perindopril arginine and Indapamide hemihydrate.

As shown in Figure 3, the linearity was achieved by plotting peak areas (y) versus the concentrations in the range of 20–450 μg/mL for Perindopril arginine and 15–112.5 μg/mL for Indapamide hemihydrate with correlation coefficients (r) ≥ 0.999. As to the calibration curve, performed in triplicate, the slopes and correlation coefficients showed high consistency, which demonstrated the reliability of the standard curve over the concentration ranges studied, as shown in Table 2.

### 3.3. Accuracy and Precision

In term of accuracy, the results were expressed as accuracy (%) of Prendopril and Indapamide in the samples. The overall results of Prendopril and Indapamide in bulk powder are demonstrated in Table 2 and Table 3, indicating the accuracy of the proposed UHPLC-UV method. 

With respect to precision, the values of %RSD for intra-day and inter-day variation are given in Table 3 and Table 4. The %RSD values in both cases were found to be acceptable within a 2% limit, indicating that the developed method is repeatable.

### 3.4. Limit of Quantification and Limit of Detection 

The LOQ was found to be 20 μg/mL for Perindopril arginine and 15 μg/mL for Indapamide hemihydrate. These concentrations gave an RSD of 1.7% and 1.6% for Perindopril arginine and Indapamide hemihydrate, respectively. However, the LOD for Perindopril arginine was found to be 6.7 μg/mL and for Indapamide hemihydrate it was 5 μg/mL, using 1 μL as an injection volume, as shown in Table 1.

### 3.5. Estimation of Perindopril Arginine and Indapamide Hemihydrate in Coversyl Plus^®^

The amounts of Perindopril arginine and Indapamide hemihydrate were indicated in triplicate using the regression equation of the pure drugs shown in Figure 3. The average amounts of Perindopril arginine and Indapamide hemihydrate present in five tablets of Coversyl Plus^®^ pharmaceutical formulation compared to the amounts claimed by the manufacturer were 4991 µg ± 0.6% and 1247 µg ± 0.84%, respectively.

### 3.6. Stability Study

**Figure 4:**
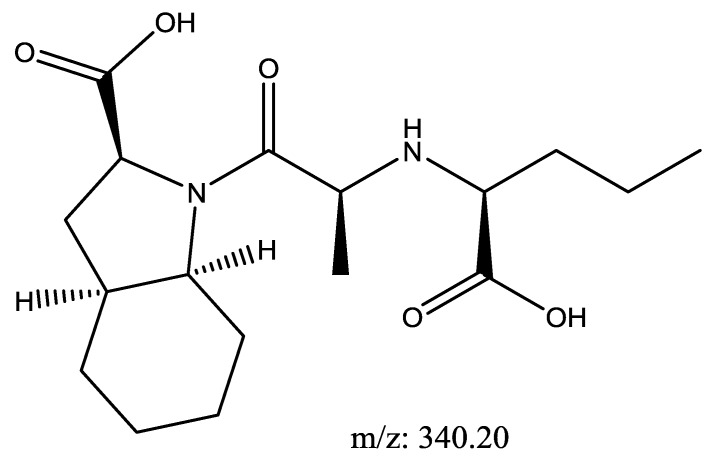
Suggested basic degradation product for Perindopril.

Perindopril with a molecular weight of 368.5 g/mol and Indapamide with a molecular weight of 365.8 g/mol were subjected to basic degradation by 1 N NaOH and heating at 90 °C for 90 min. Although the degradation product was below the detection limit on the UPLC-UV instrument, a Perindopril degradation product was identified and confirmed by UHPLC-QToF-MS analysis as in Figure 4 [28]. Indapamide was found to be stable towards basic degradation.

**Figure 5:**
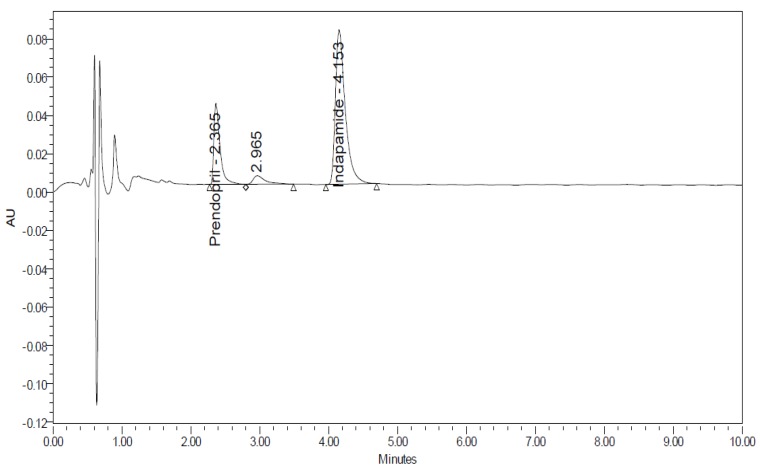
UHPLC-UV chromatogram for the acidic degradation products of Perindopril.

**Figure 6:**
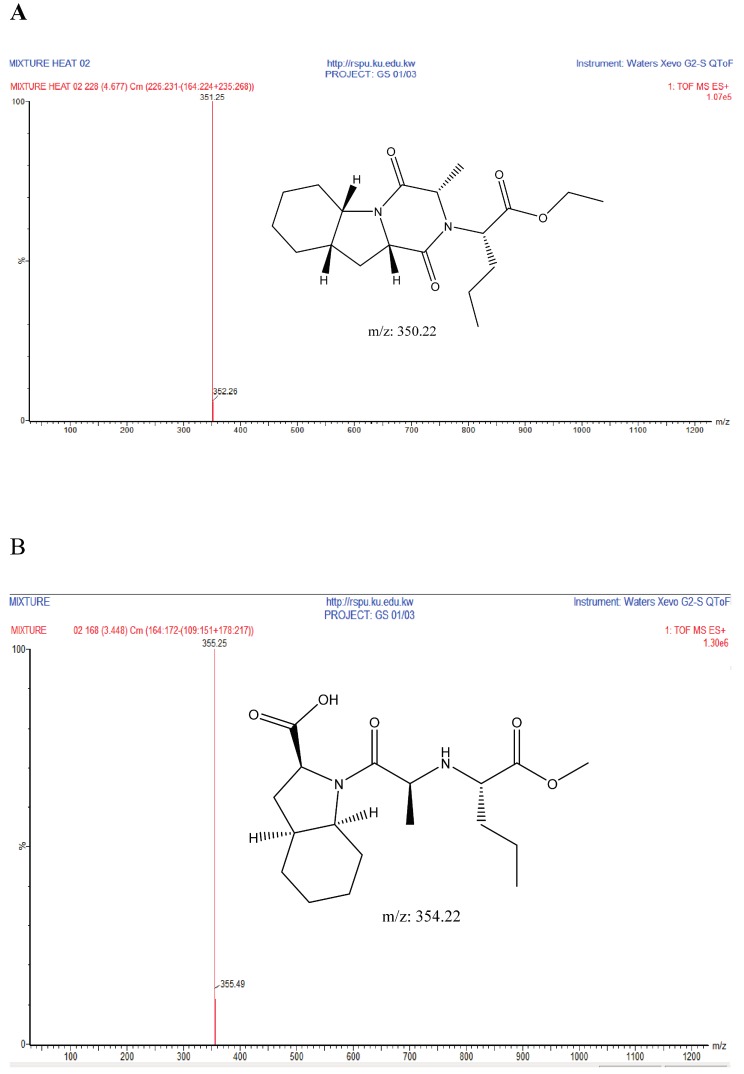

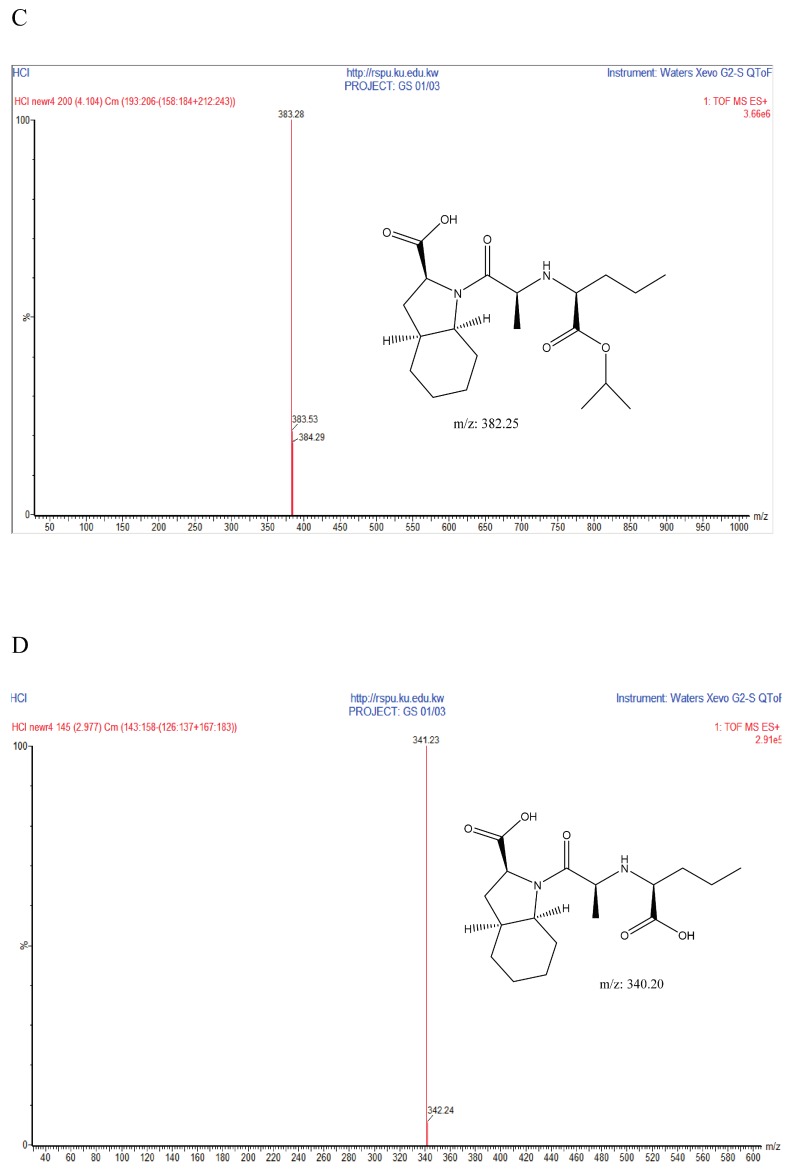
Suggested chemical structures of Perindopril degradants under acidic conditions. (**A**) Degradant A; (**B**) Degradant B; (**C**) Degradant C; (**D**) Degradant D. MS: mass spectrometry; QToF: Quadrupole Time-of-Flight.

Indapamide was found to be stable when subjected to acidic conditions by 1 N HCl; Prindopril was degraded and the degradant was eluted just after the Perindopril peak as shown in Figure 5. Moreover, the degradation product was identified by UHPLC-QToF-MS analysis and found to be degradant B. Furthermore, other degradants were detected by UHPLC-QToF-MS but their concentration was below the detection limit on UPLC-UV. The obtained *m*/*z* ratios suggested the degradation products in Figure 6 [28]. On the other hand, both analytes were found to be stable under the adopted thermal conditions.

## 4. Conclusions

In conclusion, a simple, accurate, precise, and rapid simultaneous UHPLC-UV method has been validated for the determination of a Perindopril arginine and Indapamide hemihydrate combination in pure powder and dosage form with good linearity, accuracy, and precision. The contents of Perindopril arginine and Indapamide hemihydrate in the tested pharmaceutical product (Coveryl Plus^®^: 5000 µg Perindopril/1250 µg Indapamide) were determined in triplicate and found to be 4991 µg ± 0.63 for Perindopril and 1247 µg ± 0.84 for Indapamide. Furthermore, the stability of Perindopril and Indapamide was assessed under hydrolytic and thermal conditions. Moreover, degradation products were identified by UHPLC-ESI-QToF. Both drugs were found to be stable under thermal conditions, where basic and acidic conditions resulted in Perindopril degradation.

## Figures and Tables

**Table 1:** Parameters of system suitability testing of the adopted chromatographic methods for the simultaneous determination of Perindopril arginine and Indapamide hemihydrate.

Parameters	Perindopril Arginine	Indapamide Hemihydrate	Reference Value
Resolution (R_s)_	3.584	3.584	Rs ≥ 2
Tailing factor (T)	1.55	1.81	T ≤ 2
Capacity factor (K’)	2.64	5.39	1< K’<10
Selectivity (α)	2.05	2.05	α > 1
Asymmetry factor (Af)	1.04	1.1	0.9 < Af < 1.1
Theoretical plates (N)	2268	15955	N > 2000
Height equivalent to theoretical plate (HETP; cm plate^−1^)	0.002	0.0003	The smaller the value, the higher the column efficiency

**Table 2:** Validation parameters of the proposed method.

Parameters	Perindopril	Indapamide
Range µg/mL	20–450	15–112.5
Regression Equation	*y* = 2158.5x − 4201	*y* = 13071x − 4028.5
Correlation coefficient (r)	0.9999	0.9991
LOQ (µg/mL)	20	15
LOD (µg/mL)	6.7	5
Recovery of Pharmaceutical preparation ^a^	99.81% ± 0.63	99.72% ± 0.84
Intra-assay precision ^b^	1.7	1.6
Inter-assay precision ^b^	1.9	1.8
Accuracy ^c^	97.20%	94.20%

^a^: Coversyl Plus tablets labeled to contain 5000 µg Perindopril and 1250 µg Indapamide. ^b^: expressed as the relative standard deviation (RSD). ^c^: expressed as [mean % deviation = mean calculated concentration/nominal concentration × 100].

**Table 3:** Intra-assay precision and accuracy data for Perindopril and Indapamide determination in bulk powder using UPLC-UV.

Perindopril Concentration μg/mL	Mean ± SD (*n* = 3) Observed/μg/mL	Precision ^a^ (%)	Accuracy ^b^ (%)	Indapamide Concentration μg/mL	Mean ± SD (*n* = 3) Observed/μg/mL	Precision ^a^ (%)	Accuracy ^b^ (%)
20	19.44 ± 0.331	1.7	97.20	15	14.13 ± 0.223	1.6	94.20
250	247.14±1.449	0.6	98.80	62.5	61.03 ± 0.601	1.0	97.64
450	441.67 ± 2.044	0.5	98.14	112.5	111.26 ± 0.86	0.8	98.89

^a^ expressed as the RSD. ^b^ expressed as [mean % deviation = mean calculated concentration/nominal concentration × 100].

**Table 4:** Inter-assay precision and accuracy data for Perindopril and Indapamide determination in bulk powder using UPLC-UV.

Perindopril Concentration μg/mL	Mean ± SD (*n* = 3) Observed/μg/mL	Precision ^a^ (%)	Accuracy ^b^ (%)	Indapamide Concentration μg/mL	Mean ± SD (*n* = 3) Observed/μg/mL	Precision ^a^ (%)	Accuracy ^b^ (%)
20	19.26 ± 0.373	1.9	96.30	15	13.88 ± 0.255	1.8	92.53
250	244.36 ± 1.886	0.8	97.74	62.5	58.97± 0.713	1.2	94.35
450	438.93 ± 2.453	0.6	97.54	112.5	109.91 ± 0.952	0.9	97.69

^a^ expressed as the RSD. ^b^ expressed as [mean % deviation = mean calculated concentration/nominal concentration × 100].
